# Infantile Blount disease: a developmental tibial growth defect

**DOI:** 10.11604/pamj.2021.39.74.29583

**Published:** 2021-05-26

**Authors:** Pratik Phansopkar, Shivani Uttamchandani

**Affiliations:** 1 Department of Musculoskeletal Physiotherapy, Ravi Nair Physiotherapy College, Datta Meghe Institute of Medical Sciences, Sawangi Meghe, Wardha 442001, Maharashtra, India

**Keywords:** Blount’s disease, rehabilitation, pediatrics, growth defect, physiotherapy

## Image in medicine

A 2.5 years male child, who presented with Leg bowing. As per the parents, infant started walking early at 10^th^ month and bowing of legs got worsened over time. Paediatric orthopedicians diagnosed him with endochondral disorder of the proximal end of medial tibial physis. He was referred to Physiotherapy department, for evaluation and rehabilitation. On examination, genu varum was seen (A), a positive ‘cover-up test’, internal tibial torsion, leg length difference, no tenderness, limitation of motion, and effusion, according to the review which lead to the diagnosis Blount´s disease. Lower extremity deformities in Rickets can closely mimic and osteochondro-dysplasia or genetic bone diseases which can cause lower extremity deformities similar to Blount´s disease. The metaphyseal-diaphyseal angle of bones was determined and found to be 24°, which is considered abnormal. The child´s lower leg was seen turned inward, causing the leg to appear bent below the knee (B). He had difficulties during walking and complained of pain in both the knees after 5-7 minutes of walk with lateral thrust on walking. According to Langenskiold Classification, the child is in stage 2 (2.5-4 yrs. old). As a result, parents were introduced to a home exercise program which included strengthening and stretching to correct gait and muscle imbalances, flexibility, as weak hip and core muscles contribute to knee deformities, and range of motion, which eases physical tasks and can benefit in the regression of secondary compensatory deformities, as well as Gait re-education through assistive devices (walker) improved the child´s gait parameters.

**Figure 1:**
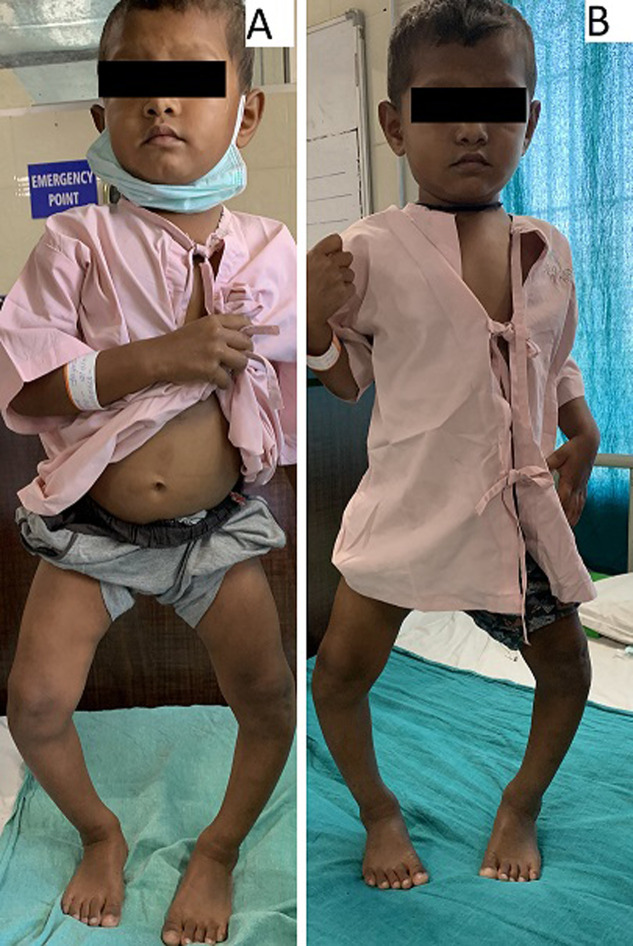
A) on examination, genu varum was seen; B) lower leg was seen turned inward, causing the leg to appear bent below the knee

